# From meta‐analysis to Cochrane reviews

**DOI:** 10.1002/jcsm.12312

**Published:** 2018-06-05

**Authors:** Nicole Ebner, Maciej Banach, Stefan D. Anker, Stephan von Haehling

**Affiliations:** ^1^ Department of Cardiology and Pneumology University Medical Center Göttingen Göttingen Germany; ^2^ Department of Hypertension, WAM University Hospital in Lodz Medical University of Lodz Poland; ^3^ Berlin‐Brandenburg Center for Regenerative Therapies (BCRT); Deutsches Zentrum für Herz‐Kreislauf‐Forschung (DZHK) partner site Berlin; Charité Universitätsmedizin Berlin Berlin Germany; ^4^ Division of Cardiology and Metabolism, Department of Cardiology Charité Universitätsmedizin Berlin Berlin Germany

**Keywords:** Meta‐analysis, Cochrane, cachexia, sarcopenia, muscle wasting

The term meta‐analysis embraces the statistical combination of results from two or more separate studies. Most meta‐analysis methods are variations on a weighted average of the effect estimates from different studies.^1^ Meta‐analyses of randomized controlled trials are usually listed as guidelines level of evidence A1. The meta‐analyses that use Bayesian modelling to incorporate direct trial evidence and indirect evidence to provide a more precise estimate of the treatment effects (network meta‐analyses [NMA]) and especially those based on the individual patients' data from the studies have obviously the largest impact. Cochrane reviews are systematic reviews of primary research in human health care and are internationally recognized as the highest standard of evidence‐based health care resources. Cochrane reviews are thus systematic reviews that appraise and analyse evidence according to a priori defined criteria. The methods that were used in trials, the types of interventions, the patient population, and outcome measures are all assessed in Cochrane reviews. The principal part of Cochrane systematic reviews is that they are regularly updated (approximately every 2 years) and that they are published online in the Cochrane Library.

## Historical development

As early as 1972, the British epidemiologist Archibald Cochrane (1909–1988) had observed that in many medical decisions, the academic knowledge had often been disregarded because the medical literature had become too large and complex.^2^ In 1979, Cochrane wrote:

It is certainly a great failure of our profession that we have not organized a critical summary of all randomized controlled trials, which is sorted by specialty or subspecialty and is updated regularly.^3^

His idea fell on fertile ground. In Oxford, a group of doctors and methodologists began to gather in the field of obstetrics on the main issues and summarized all randomized trials using the meta‐analysis approach.^4^ Cochrane praised this pilot project shortly before his death as a milestone in medical research. From one of these meta‐analyses, the logo of the Cochrane Collaboration was developed later.^1^ Funded by the British health care system, the first Cochrane Centre was opened in Oxford in October 1992. The Cochrane Collaboration has since grown steadily.^1^ It is an international network, which is free of financial interests. Its declared aim is to create, update, and disseminate systematic reviews on health care issues. Similarly to any medical and scientific journal, the Cochrane group exist of one head of the Review Group surrounded by a group of experts or section editors. The group of editors that examine the Cochrane reviews are similar to section editors and finally the reviewers themselves who create a single scientific issue a systematic review of the manuscript. While the core of the Review Group, called the Editorial Base, is mostly localized in one place, the reviewers might be from all over the world and collaborate internationally. These electronic channels of communication are almost used exclusively.

## The Cochrane review

As a first step to a Cochrane review, authors can register its content with the competent Review Group. This prevents that a second author can start working on the same subject. After that, a protocol of the review is created, which is audited by two experts in most cases. In order to ensure the safe handling of statistical meta‐analysis, a special team within the Cochrane Collaboration has developed dedicated computer software. The program ‘Review Manager’, abbreviated ‘RevMan’, is freely available on the Internet.^1^ In the program, there are several statistical measures, models, and graphics available, for example, the funnel plot. There is also strongly recommended the program manual that can be used as a guide for creating a meta‐analysis. As only the review is completed, it is next examined by the evaluators of the Review Group and finally included in the Cochrane Library. Once crucial new data are published, there is a need to update the review.

## The Cochrane Library

The Cochrane Library itself has an impact factor of about 6.124, and it usually contains the Cochrane reviews and review protocols. In addition, there is a second important database containing more than 350 000 references with controlled clinical trials. The advantage of this study registry is that only studies are available that are deemed relevant to the clinician, but these of the highest integrity.

## Executive summary of Cochrane reviews

As editors of the Journal of Cachexia, Sarcopenia and Muscle, we invited authors of published Cochrane reviews to write executive summaries of their Cochrane reviews with topics focusing on cachexia, sarcopenia, and muscle. Four authors have accepted to write such summaries for our Journal. And the first summary of Cochrane review was printed in the September 2015 issue. Grande *et al*.^5^, ^6^ showed the relevance of exercise for cancer cachexia in adults. This review was of special interest because the authors screened 3154 separate titles and abstracts and reviewed 16 full‐texts. They sought randomized controlled trials in adults meeting international criteria for cancer cachexia, comparing a program of exercise as a sole or adjunct intervention to usual care or an active control. CENTRAL, MEDLINE, EMBASE, DARE and HTA, ISI Web of Science, LILACS, PEDro, SciVerse SCOPUS, Biosis Previews PreMEDLINE, and Open Grey databases were searched up to June 2014.^4^, ^5^ Two authors independently assessed studies for eligibility. Additionally, corresponding authors were contacted to determine if samples met cachexia staging criteria. Most authors did not explore this concept. Finally, no trial met review eligibility criteria; therefore, they were unable to perform a meta‐analysis to determine any effects from exercise intervention.^4^, ^5^ This impressively showed the need of trials in cachexia and exercise testing.

The executive summary by Mücke *et al*. was the second Cochrane review that was published in Journal of Cachexia, Sarcopenia and Muscle in March 2016.^7^, ^8^ The aim of the Cochrane review was to evaluate the efficacy of pharmacological treatments for fatigue in palliative care, with a focus on patients at an advanced stage of disease, including patients with cancer and other chronic diseases. A total of 1645 publications were selected during the literature search. They identified 45 studies for inclusion, with a wide range of underlying diseases and drug interventions. Based on that they showed that there is insufficient evidence to support the use of a specific medicine to treat fatigue in palliative care patients. In this regards, amantadine showed the promised benefit in patients with multiple sclerosis with fatigue and methylphenidate in patients with cancer‐related fatigue.^6^, ^7^

Connolly *et al*. wrote the third Cochrane executive summary that was published in December 2016.^9^, ^10^ They aimed to determine the effectiveness of exercise rehabilitation initiated after intensive care unit discharge on primary outcomes of functional exercise capacity and health‐related quality of life. A total of 4298 results, of which 276 underwent title and abstract screening, were identified. After selection, six completed and fully published trials indicating an expanding evidence base for this clinical field. Consequently, they were unable to confirm the efficacy of post‐intensive care unit discharge exercise‐based rehabilitation on the selected outcomes. Most included studies failed to show a significant difference between the intervention and the control groups. The degree of heterogeneity across included studies precluded a meta‐analysis of data, and individual study findings were inconsistent with regard to beneficial effects on functional exercise capacity.^9^, ^10^

Despite the continuous debate on the real effect of meta‐analysis on recommendations as well as on their limitations, meta‐analysis is more and more in the focus of guideline committees and clinicians, and we are happy that our Journal published several important meta‐analyses on relevant topics in recent years.^11^, ^12^, ^13^ Meta‐analyses of randomized controlled trials have the highest levels of evidence in the guidelines. But there is still a question on whether there is a hierarchy of evidence in using meta‐analysis. Because meta‐analyses are a key base for evidence‐based medical care, their creation should meet the highest methodological standards.^1^ At the same time, there are still many topics in medicine without any meta‐analyses available that would be highly desirable. We therefore kindly welcome submissions of executive summaries of Cochrane reviews or well‐conducted meta‐analyses.

## Conflict of Interest

None declared.
